# Activated Complement Factors as Disease Markers for Sepsis

**DOI:** 10.1155/2015/382463

**Published:** 2015-09-02

**Authors:** Jean Charchaflieh, Julie Rushbrook, Samrat Worah, Ming Zhang

**Affiliations:** ^1^Department of Anesthesiology, Yale University School of Medicine, New Haven, CT 06510, USA; ^2^Department of Anesthesiology, College of Medicine, SUNY Downstate Medical Center, Brooklyn, NY 11203, USA; ^3^Department of Cell Biology, College of Medicine, SUNY Downstate Medical Center, Brooklyn, NY 11203, USA

## Abstract

Sepsis is a leading cause of death in the United States and worldwide. Early recognition and effective management are essential for improved outcome. However, early recognition is impeded by lack of clinically utilized biomarkers. Complement factors play important roles in the mechanisms leading to sepsis and can potentially serve as early markers of sepsis and of sepsis severity and outcome. This review provides a synopsis of recent animal and clinical studies of the role of complement factors in sepsis development, together with their potential as disease markers. In addition, new results from our laboratory are presented regarding the involvement of the complement factor, mannose-binding lectin, in septic shock patients. Future clinical studies are needed to obtain the complete profiles of complement factors/their activated products during the course of sepsis development. We anticipate that the results of these studies will lead to a multipanel set of sepsis biomarkers which, along with currently used laboratory tests, will facilitate earlier diagnosis, timely treatment, and improved outcome.

## 1. Introduction

Sepsis is the third most common cause of death in the United States [1] and among the top 10 causes of death worldwide [2, 3]. The incidence of sepsis is increasing due to multiple factors, including the aging of the population, the performance of more invasive procedures, and the continuing emergence of antibiotic-resistant microorganisms [4]. Evidence- and protocol-based management of sepsis, such as that recommended in the Surviving Sepsis Campaign guidelines [5, 6], has been shown to be effective in improving outcomes but remains a challenge in resource-limited settings [7, 8]. The traditional emphasis on early recognition and management in the hospital setting is now being extended to the prehospital setting as recent evidence demonstrates that sepsis is a challenging problem there. A recent study of 407,176 emergency medical service (EMS) encounters found that the incidence of severe sepsis was 3.3 per 100 EMS encounters, notably greater than that for acute myocardial infarction and stroke (2.3 and 2.2 per 100 EMS encounters, resp.) [9]. Improvement in the clinical care of sepsis requires (1) identifying markers that are associated with early stages of sepsis and (2) identifying markers that correlate with established stages of sepsis. Disease markers can guide the delivery of evidence-based therapies for sepsis which will include timely administration of antibiotics, infection-source control, provision of intravenous fluids, vasoactive agents and immune response-modification, and supportive therapy including mechanical ventilation [10, 11].

In order to identify effective disease markers for sepsis, it is necessary to understand the mechanisms involved in sepsis development. In general, the sepsis inflammatory response starts with changes in intracellular structures, particularly the mitochondria and the cytoskeleton [10]. This is followed by release of various inflammatory mediators to the extracellular milieu leading to a hyperinflammatory state, accompanied by a buildup of oxidants in tissues indicating an imbalance between reduction and oxidation processes [12]. Among the factors in the hyperinflammatory response, circulating complement factors are recognized as playing an important role [12, 13]. These factors contribute to the development of clinical symptoms observed in patients' sepsis such as fever and hemodynamic instability [3].

In this review, we present the latest findings regarding individual complement factors involved in the pathogenesis of sepsis together with their potential value as disease markers and discuss new results from our laboratory concerning the involvement of mannose-binding lectin (MBL) in patients with septic shock. Overall, accumulating evidence suggests that changes in the levels of certain complement factors or their activated products reflect the course of sepsis and may thus serve as potential staging markers.

## 2. Brief Review of Complement Pathways

**Figure 1:**
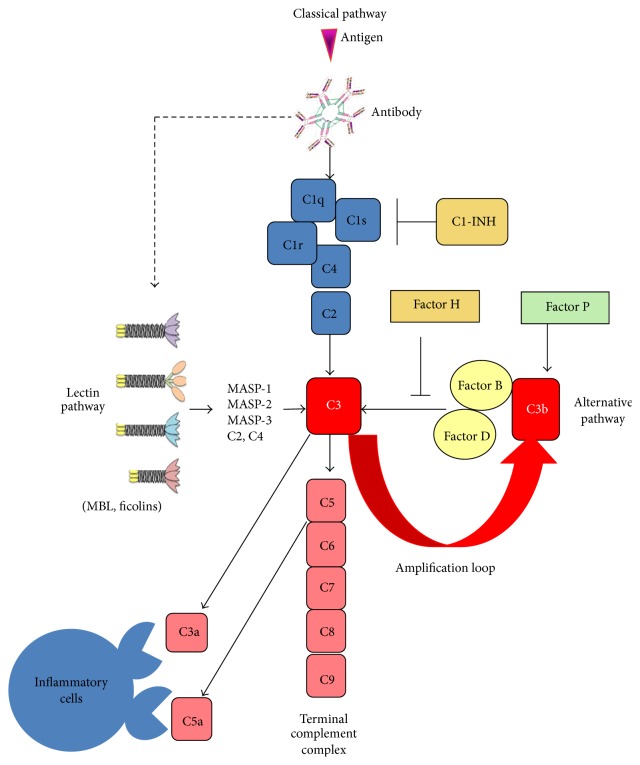
The pathways of activation of the complement system.

The complement system, which consists of multiple proteins in body fluids, receptors, and regulatory proteins, defends against infectious agents and acts as an immune effector and regulator. Complement activation, in general, can be initiated via three pathways (Figure 1): the classical pathway (including antibodies, C1q, C2, and C4), the alternative pathway (including complement factor B (CFB) and spontaneous C3 hydrolysis to form C3b), and the lectin pathway (including MBL and ficolins) [14–21]. These pathways, which proceed with sequential activations of proteases, converge at the formation of C3 convertase (C3b•Bb) which acts on intact C3 to generate more C3b and forms C5 convertase (C3b•Bb•C3b) which acts on intact C5 to continue in the common complement pathway. The common complement pathway results in formation of the terminal complement complex, that is, the membrane attack complex (MAC, C5b•C6•C7•C8•C9). MAC binds to and destroys cellular targets. Amplification via a loop involving C3 convertases occurs only through the CFB-dependent alternative pathway [22, 23]. The C3 and C5a convertases are regulated by membrane-bound proteins (e.g., CD35, CD46, and CD55) and soluble factors (e.g., factor H and factor I) [22, 24].

## 3. The Involvement of Complement C4 of the Classical Pathway in Sepsis

Complement factor C4, along with C1q and C2, functions in the classical complement pathway by forming C3 convertase (C4bC2a) and contributes to activation of C3. C4 can also function in the lectin pathway, where it is activated by any of the three MBL-associated serine proteases (MASPs) again leading to activation of C3. The involvement of the initial factors of the classical complement pathway in sepsis, antibodies, and complement C1q has been reviewed elsewhere [25]. This review will focus on recent reports of the role of complement C4.

An early clinical study of 20 patients with sepsis showed that uncleaved C4 remained unaltered between admission and 96 h later, despite decreased total complement activity as measured by the 50% hemolytic complement (CH_50_) assay [26]. In the same study, 19 patients who were in septic shock had markedly decreased levels of uncleaved C4 together with decreased total complement activity. After 96 h, the decreased values returned to the normal range. A more recent study of 21 patients with sepsis showed that, on day 1 after intensive care unit (ICU) admission, nonsurvivors had significantly lower levels of uncleaved C4 than survivors, but that the C4 levels of the two groups became similar after three days in the ICU [27]. Supporting this finding, a recent study of a larger number (76) of septic patients found increased C4 activation and thus C4 consumption in sepsis [28]. Further studies with large numbers of septic patients are needed to determine if the uncleaved C4 level can be used as a disease marker for sepsis. If the uncleaved C4 level at admission is found to predict the survival outcome of septic patients, it could be used as a staging biomarker for sepsis.

## 4. The Involvement of Complement Factor B (CFB) of the Alternative Pathway in Sepsis

CFB functions in the alternate pathway to activate and amplify the complement system [22, 23]. A recent mouse septic shock study indicated that the absence of CFB conferred a protective effect, with improved survival and cardiac function and markedly attenuated acute kidney injury [29]. Activation of Toll-like receptors (TLR2, TLR3, and TLR4) markedly enhanced CFB synthesis and release by macrophages and cardiac cells. This suggested to the researchers that CFB, acting outside of the alternative pathway, is a downstream effector of Toll-like receptors (TLRs) and plays an important role in the mouse model of severe sepsis. Contrary to the conclusion of this study, a number of clinical studies indicated that the alternative complement pathway is essential in the fight against infection and is activated in clinical settings of septic shock [30–33]. Clinical studies showed that the active fragment of CFB, Bb, as well as the ratio Bb/CFB, was significantly increased in septic shock patients [34, 35]. Future studies are needed to examine the time course of Bb changes during septic shock progression in humans to determine if Bb can serve as a staging marker of sepsis.

## 5. The Involvement of Factors of the Lectin Complement Pathway in Sepsis

### 5.1. MBL

MBL has been the most studied of the initial factors in the lectin complement pathway [14–18, 20]. MBL circulates bound with any one of three MASPs [36, 37]. When bound to certain carbohydrate patterns on pathogens, MBL activates its bound MASP, which in turn cleaves C4 and C2 to form the C3 convertase [38–40]. An early clinical study suggested that deficiency of MBL function was associated with bloodstream infection and the development of septic shock [41]. Risk of infection and sepsis in severely injured patients were found to be related to single nucleotide polymorphisms in the genes for proteins in the lectin pathway: a variant of MBL2 contained an exon 1 nucleotide change; a MASP2 variant contained the amino acid change Y371D; and a ficolin 2 variant contained the amino acid change A258S [42]. In critically ill patients, higher incidence and a worse prognosis of severe sepsis/septic shock appear to be associated with low-producer haplotypes of MBL [43]. However, there are controversial results regarding MBL's involvement in sepsis. Two septic shock patients with MBL deficiencies were found to have relatively low disease severity and mild DIC compared with 16 septic shock patients who were MBL-sufficient [44]. In agreement with the latter finding, a more recent study of 267 septic patients found that MBL levels were higher in the septic patients with DIC than in those without DIC [45]. In a study of 128 patients with sepsis and septic shock, the majority of patients did not have changes in MBL on days 1, 3, 5, and 7 after diagnosis [46]. A recent clinical study also found that MBL deficiency did not influence complement activation in asymptomatic HIV infection and HIV-infected patients with sepsis or malaria [28].

We investigated the temporal blood levels of MBL in 16 patients after septic shock diagnosis and admission and the correlation of MBL levels with in-hospital mortality. Our preliminary results showed that MBL levels did not change significantly with all patients analyzed in one group in the 5-day interval after diagnosis of septic shock (supplementary Figure 1a in Supplementary Material available online at http://dx.doi.org/10.1155/2015/382463). In addition, there were no significant differences in MBL values between MBL survivors and nonsurvivors at time points 6, 24, 48, 72, and 96 hrs after diagnosis of sepsis. Compared with the zero time point (time of diagnosis of sepsis) values, there was a trend which was not statistically significant in that survivors had an increase in MBL between 1 and 3 days after diagnosis during the 5-day observation period, while the nonsurvivors had a smaller increase between 2 and 3 days (supplementary Figure 1b). Taken together with published reports, these results suggest that MBL would not be a good staging or monitoring marker for sepsis.

### 5.2. Ficolins

There are 3 types of ficolins in humans, L-, H-, and M-ficolin (also referred to as ficolin-2, ficolin-3, and ficolin-1, resp.). Like MBL, ficolins recognize certain carbohydrate patterns on pathogens. Following this recognition, MASPs are activated and the C3 convertase formed [38–40]. Recent research suggested that a high M-ficolin level in neonatal cord blood (>1,000 ng/mL) was associated with early-onset sepsis in newborns [47]. A recent murine study showed that ficolin-B, the mouse orthologue of human M-ficolin, was stored in and released from immature granulocytic myeloid cells during sepsis [48]. If future larger studies confirm the predictive value of M-ficolin for early onset of sepsis, M-ficolin has potential for use as a diagnostic or staging marker. The temporal profiles of the serum L- and H-ficolins in human sepsis are unknown. An* in vitro* study showed that L-ficolin in cord serum functioned synergistically with capsular polysaccharide-specific IgG to bring about opsonophagocytic killing of the bacterium* Streptococcus* [49]. Further studies are needed to investigate the roles and potential diagnostic applications of ficolins in sepsis.

## 6. Factors of the Common Complement Pathway Involved in Sepsis

### 6.1. Complement C3

C3 is the central factor in the complement system and can be activated by the classical, the lectin, and the alternative pathways of complement. Activation of C3 in turn activates the downstream common terminal pathway. C3 deficiency leads to increased susceptibility to infection [50–52]. C3^−/−^ mice had significantly reduced survival in septic shock models [53, 54]. Surprisingly, C3^−/−^ mice developed the full intensity of acute lung injury (ALI) in a C5a-dependent manner due to the action of thrombin that generates C5a directly from C5 [55]. Exogenous C3 administration markedly improved the 48-hour survival rate in a recent study of polymicrobial sepsis in wild type mice [56].

In patients that have suffered burn injuries, recent clinical studies have shown that uncleaved C3 levels were inversely correlated with the severity of burn and could be used to predict the onset of infection, septicemia, and mortality [57]. Depletion of uncleaved C3 was also found to be linked to the expansion of T-regulatory cells during abdominal sepsis and to be an indicator for prolonged hospital stay and poor prognosis [58, 59]. However, a study of 267 septic patients found that uncleaved C3 levels were higher in the DIC group [45]. In other studies the activated C3 fragments, C3a and C3b/c, were elevated in septic shock patients and correlated with mortality [60–65]. The increase in C3a levels occurred at day 1 after diagnosis [66]. However, the details of the time course of the C3a formation in the blood have not been determined. Overall, the majority of the studies suggest that C3 is essential for control of bacteremia and is a potential monitoring marker for sepsis. Further studies are needed to determine if the C3a level can be used as a disease marker for sepsis.

### 6.2. Complement C5 and the Membrane Attack Complex (MAC)

C5 is important not only for formation of the terminal complement complex MAC, but also for attracting inflammatory cells via its fragment C5a, which is a potent chemoattractant. C5a is also an anaphylatoxin, mediating inflammation by inducing mast cell degranulation and histamine release. A deficiency in C5 has been associated with increased risk of recurrent* Neisseria* infections [67]. A recent study of 60 septic shock patients found significantly increased serum levels of C5 activation products C5a and MAC [60].

There are two known receptors for C5a, namely, C5aR and C5L2 (the most recent nomenclatures for these are C5aR1 and C5aR2, resp.) [55, 68]. Neutrophils from patients in septic shock exhibited decreased C5aR expression, levels of which correlated inversely with serum concentrations of C-reactive protein (a protein produced in the liver that increases in plasma during inflammation [69]) and a positive clinical outcome [60]. Animal studies using knockout mice for the second C5a receptor, C5L2, produced different results depending on the animal model [70]. C5L2-deficient mice were hypersensitive to septic shock in a lipopolysaccharide- (LPS-) induced model [71]. Unlike C5aR, C5L2 is believed to be a recycling decoy receptor [72] which physically interacts with both C5aR and *β*-arrestin to negatively regulate C5aR signaling in an anti-inflammatory manner and to reduce pathology [73]. In contrast, other investigators, particularly Ward's group, showed that blockade or absence of either of the C5a receptors, C5aR and C5L2, improved survival and attenuated the buildup of proinflammatory mediators in plasma in a mouse sepsis model of cecal ligation and puncture (CLP) [74]. A follow-up study further showed splenocyte apoptosis and significant lymphopenia 3 days after initial CLP in wild-type mice but not in C5aR^−/−^ or C5L2^−/−^ mice [75]. C5L2 stimulation by C5a caused release from cells of the protein high mobility group box 1 (HMGB1) both* in vitro* and* in vivo* and enhanced pathology in sepsis models [74]. HMGB1 is a late mediator of sepsis released by macrophages/monocytes in response to pathogen-associated molecular patterns [76].

The activated C5 fragment, C5a, modulates intracellular signaling pathways such as ERK1/2 signaling in macrophages via heteromer formation with C5aR/C5L2 and *β*-arrestin recruitment [77]. This modulation of ERK1/2 activation may be the mechanism by which C5a receptor heteromers regulate cytokine release. C5a enhances granulocyte-colony stimulating factor (G-CSF) production in urokinase-type plasminogen activator expression in macrophages [78, 79]. In addition, C5a regulates the migration of IL-12^+^ dendritic cells to induce the development of pathogenic Th1 and Th17 cells in sepsis [80]. Recent studies showed that there is crosstalk between C5 activation and other pathways [81], particularly TLR pathways [82]. Blocking C5 and the protein receptor cluster of differentiation 14 (CD14), which binds TLR-4, abolishes the inflammatory response and improves survival in sepsis models [83–86].

C5a can be regulated by the protein nucleotide-binding oligomerization domain containing 2 (NOD2), an intracellular sensor for small peptides derived from the bacterial cell wall components present in antigen-presenting cells (APCs) and epithelial cells [87]. In a CLP-induced model, NOD2 mediates suppression of expression of the protein CD55 on the surface of neutrophils and enhances C5a generation during polymicrobial sepsis [88]. The C5aR receptor is regulated by the neutrophil serine protease (NSP), which cleaves it in response to excess C5a generation or necrosis [89].

A recent study found that a serum-circulating form of C5aR (cC5aR) may represent a new sepsis disease marker to be considered in tailoring individualized immune-modulating therapy [60]. cC5aR was detected in septic patients' serum at the time when the patients required continuous infusion of vasopressors or inotropic agents to maintain blood pressure, despite adequate fluid resuscitation. In an animal CLP model, levels of cC5aR began to increase immediately after sepsis induction, peaking after 12 h. Between 12 and 24 h, the cC5aR concentration rapidly declined [60]. It remains to be determined whether the levels of cC5aR change in a similar manner in humans. If they do, it would be valuable to know whether cC5aR levels change as effective clinical interventions are implemented. Such changes would suggest that cC5aR could be used as a monitoring marker to determine the effectiveness of a therapy and to correlate it with clinical outcome.

The MAC were found to be higher in the septic patients with DIC than those without DIC, and the MAC was an independent predictor of sepsis-induced DIC [45]. The increase in MAC levels occurred at day 1 after diagnosis [66].

## 7. Complement Regulators in Sepsis

### 7.1. Properdin

Properdin (Factor P) is the only known positive regulator of complement activation. As serum protein, it increases the production of complement activation products in the alternative pathway by binding C3b present in the cell membrane-attached C3 convertase complex, C3b•Bb, and stabilizing the complex [90]. A recent animal study showed that properdin-deficient mice had increased survival rates in a streptococcal pneumonia model of sepsis [91]. In contrast, a low-dose of a recombinant properdin (more effective than native properdin in promoting complement activation via the alternative pathway) provided substantial protection against both* Streptococcus pneumoniae* (*S. pneumonia*) and* Neisseria meningitides* (*N. meningitides*) infections [92]. A recent clinical study showed that the properdin levels in serum from 81 critically ill patients (with predominately abdominal or respiratory sepsis) were significantly decreased at time of admission to the intensive care unit (ICU) but increased after clinical recovery to exceed levels observed in healthy volunteers [93]. Among the septic patients, properdin concentrations at ICU admission were decreased in nonsurvivors of sepsis compared to survivors. Further, pathologically low properdin levels were related to increased duration of treatment. Based on this study, a decreased level of properdin may be a diagnostic marker for the initial stage of sepsis, and the increase in properdin levels after treatment may be a staging marker.

### 7.2. Factor H

Factor H is a negative regulator of amplification through the alternative pathway [94–98].* In vitro* studies revealed that bacteria such as* N. meningitidis* and* S. pyogenes* recruit factor H to their surfaces via a factor H binding protein, providing a mechanism to avoid host complement-mediated killing [95, 96]. The temporal profile of serum factor H in human sepsis conditions is unknown.

## 8. Discussion and Conclusions

While the cumulative evidence from animal and clinical studies regarding the involvement of complement factors in sepsis is incomplete, it is clear that complement activation is prominent in the events leading through sepsis to septic shock [99]. The activated fragments in the terminal common pathway of complement, that is, C3a, C5a, and the soluble form of the C5a receptor, cC5aR, may be useful as monitoring markers which reflect the effectiveness of therapy and correlate with clinical outcome.

Recent clinical studies showed that C4 levels in the classical pathway decreased in the early stages of sepsis and may correlate with survival. Regarding the alternative pathway, CFB was reported to be activated and its Bb fragment levels increased in septic patients. Among the initiators of the lectin pathway, cumulative evidence suggests that MBL is not a good staging or monitoring marker for sepsis. While there are relatively few studies of the other initial lectin factors in sepsis, namely, the M-, L-, and H-ficolins, a recent report suggested that cord-blood M-ficolin levels may correlate with early onset of sepsis in newborns. Among the regulatory proteins of complement, properdin was found to be decreased at the initial stage of sepsis and increased after effective treatment; thus it may have potential as a staging marker. Most of these clinical studies enrolled a small number of patients. Further research with large subject numbers is needed to confirm the usefulness of particular complement factors as markers for sepsis.

Currently, work in clinical settings has not yielded the requisite gold standards for diagnosis and monitoring of sepsis. Advances in systems biology and other new technologies have the potential to identify these [100]. Microarrays have been used to screen for differentially expressed genes related to severe sepsis induced by multiple trauma [101]. Pyrosequencing of DNA, which reads short sequences, could lead to rapid identification of infectious agents, allowing targeted selection of antibiotics and improvement of patients' prognoses [102]. Mass spectrometry-based proteomics is a powerful tool and has been applied to serum and urine for identification of biomarkers for sepsis. Many potential biomarker candidates have been identified, including altered levels of complement factors [103, 104]. Validation of the clinical use of these biomarker candidates may significantly impact the diagnosis and prognosis of sepsis.

Although the scientific approaches described above have the potential to provide information that will improve diagnostic ability, prognosis assessment, therapeutic target identification, and treatment stratification, the approaches themselves are expensive and time-consuming and are not useful as point-of-care tests in the emergency setting. In contrast, blood tests for biomarkers remain indispensable [105]. With regard to complement factors as potential biomarkers, clinical studies, assisted as necessary by the new technologies discussed above, are needed to obtain the complete temporal profiles of activation of complement factors in the course of sepsis development. We anticipate that the results will provide a multipanel set of complement factor biomarkers, which can be coupled with routine lab tests, for example, neutrophil-lymphocyte count ratio [106] and blood culture, for the staging and monitoring of events in sepsis. These efforts would facilitate earlier diagnosis, timely treatment, and informed prognosis.

## Supplementary Material

Sixteen patients with diagnosis of septic shock were enrolled in this study. Peripheral blood was collected in 5-day interval after diagnosis of septic shock and the levels of MBL were determined by ELISA. Comparisons of the MBL levels in the subgroups and in-hospital mortality were analyzed by SPSS software.
