# Supplementary figures and images for: Activated Complement Factors as Disease Markers for Sepsis
Source: Dis Markers. 2015 Sep 2;2015:382463. doi: 10.1155/2015/382463 (PMC4572436; doi:10.1155/2015/382463)

Supplement Figure 1

*a*

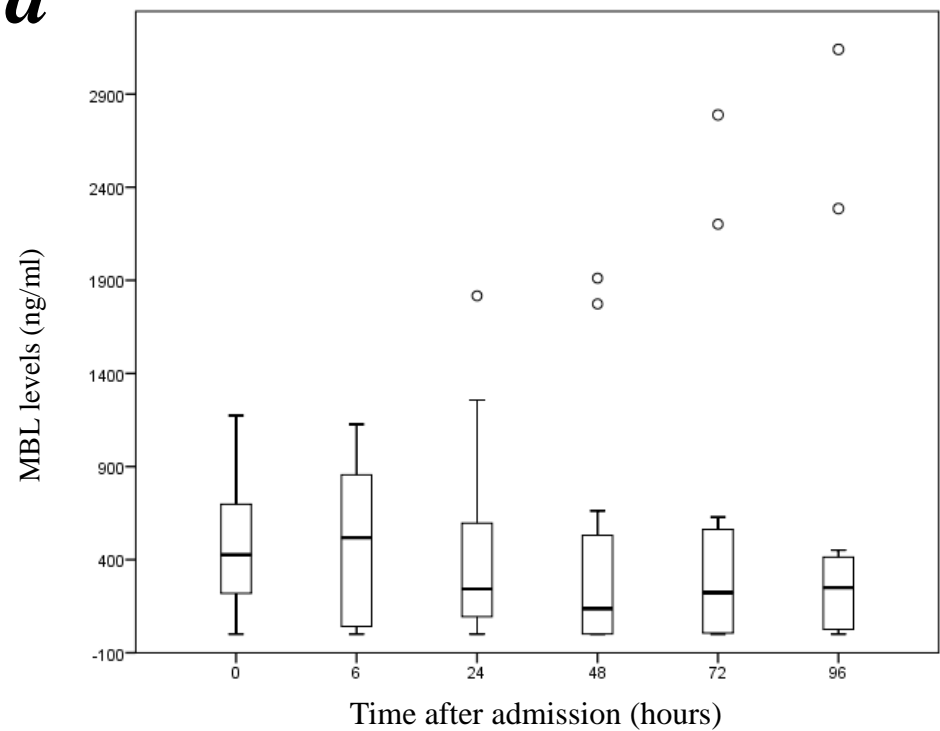

*b*

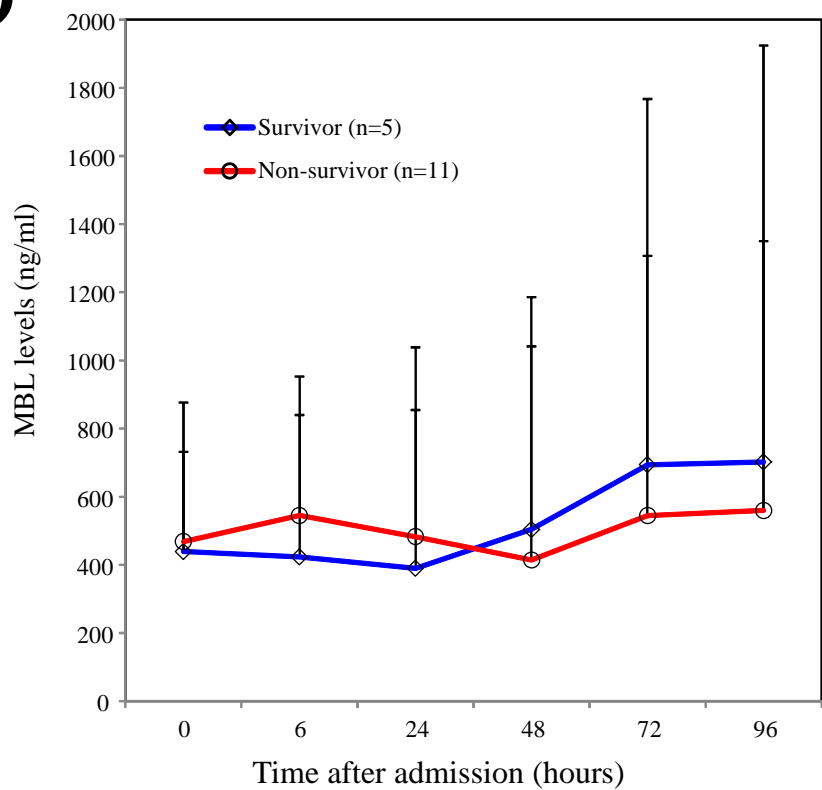

Supplement: Supplementary file 1 — Sixteen patients with diagnosis of septic shock were enrolled in this study. Peripheral blood was collected in 5-day interval after diagnosis of septic shock and the levels of MBL were determined by ELISA. Comparisons of the MBL levels in the subgroups and in-hospital mortality were analyzed by SPSS software. [file 382463.f1.pdf]
